# Exosomes in Alzheimer’s Disease: From Being Pathological Players to Potential Diagnostics and Therapeutics

**DOI:** 10.3390/ijms221910794

**Published:** 2021-10-06

**Authors:** Hagar M. Soliman, Ghada A. Ghonaim, Shaza M. Gharib, Hitesh Chopra, Aya K. Farag, Mohamed H. Hassanin, Abdalrazeq Nagah, Mahmoud Emad-Eldin, Nevertary E. Hashem, Galal Yahya, Sherif E. Emam, Abdalla E. A. Hassan, Mohamed S. Attia

**Affiliations:** 1 Department of Pharmaceutics, Faculty of Pharmacy, Zagazig University, Zagazig 44519, Egypt; ha.8888ms@gmail.com (H.M.S.); ghada99mahmoud@gmail.com (G.A.G.); shathamahmoudelbahnasawy@hotmail.com (S.M.G.); ayak3326@gmail.com (A.K.F.); m.hassan131415@gmail.com (M.H.H.); Abdalrazeqnagah@gmail.com (A.N.); nevertary360@gmail.com (N.E.H.); sherif.emam86@yahoo.com (S.E.E.); 2 Chitkara College of Pharmacy, Chitkara University, Punjab 140401, India; chopraontheride@gmail.com; 3 Department of Clinical, Faculty of Pharmacy, Zagazig University, Zagazig 44519, Egypt; mahmoud.emad927@yahoo.com; 4 Department of Microbiology and Immunology, Faculty of Pharmacy, Zagazig University, Zagazig 44519, Egypt; galalmetwally2020@gmail.com; 5 Applied Nucleic Acids Research Center & Chemistry, Faculty of Science, Zagazig 44519, Egypt; habdallaa@aol.com

**Keywords:** exosomes, neurodegenerative disorders, drug delivery, diagnosis, treatment, cargo, microfluidics

## Abstract

Exosomes (EXOs) were given attention as an extracellular vesicle (EV) with a pivotal pathophysiological role in the development of certain neurodegenerative disorders (NDD), such as Parkinson’s and Alzheimer’s disease (AD). EXOs have shown the potential to carry pathological and therapeutic cargo; thus, researchers have harnessed EXOs in drug delivery applications. EXOs have shown low immunogenicity as natural drug delivery vehicles, thus ensuring efficient drug delivery without causing significant adverse reactions. Recently, EXOs provided potential drug delivery opportunities in AD and promising future clinical applications with the diagnosis of NDD and were studied for their usefulness in disease detection and prediction prior to the emergence of symptoms. In the future, the microfluidics technique will play an essential role in isolating and detecting EXOs to diagnose AD before the development of advanced symptoms. This review is not reiterative literature but will discuss why EXOs have strong potential in treating AD and how they can be used as a tool to predict and diagnose this disorder.

## 1. Introduction

Alzheimer’s disease (AD) is a prevailing disorder among the elderly, accounting for more than 60% of dementia cases, which may be either sporadic or familial, representing the late and early onset of the disease, respectively. However, sporadic is more common, corresponding to over 90% of AD cases. AD is linked to a set of memory and cognitive disabilities that progress along the disease course, including primarily minor loss of short-term memory, which advances to mood swings, agitation, irritability and aggressive behavior, sequential aphasia, and long-term memory loss, followed by a moderate reduction in vital functions [1,2,3].

The presence of amyloid-beta (Aβ) plaques and neurofibrillary tangles (NFTs) in the brain autopsies of AD patients revealed them as the critical players in the pathophysiology of AD [4]. The handling of amyloid precursor protein (APP) by secretases gives rise to extracellular Aβ monomers, which adhere to form clumps or groups of Aβ plaques. These plaques stimulate the immune system to generate an inflammatory response that damages the surrounding neurons [5]. Further, defective hyperphosphorylation and misfolded tau proteins yield intracellular NFTs and impair signaling across the neurons [6].

FDA-approved medications fail to offer a definitive treatment of AD pathophysiology; however, they provide symptomatic aid in delaying the progression. AD treatment generally shares one target by increasing the Acetyl Choline level by preventing its degradation and thus preserving intact memory and cognitive functions [7]. New trends in treatment, such as disease-modifying AD therapies, have emerged to address the disadvantages of conventional therapeutics. However, this approach requires comprehensive knowledge of metabolic pathways and harnesses various targeting strategies to provide a successful medication [4].

Exosomes (EXOs) were discovered as extracellular vesicles released upon the exocytosis of a multivesicular body (MVB) [8]. The biogenesis of EXOs is a sequential process of plasma membrane budding, where inward budding “endocytosis” forms an early endosome, which matures to generate a late endosome or MVB. Proteins, micro-RNAs, and messenger RNAs invaginate the MVB, forming intraluminal vesicles, fusing with the cell membrane, releasing EXOs [9].

As a dual role player, EXOs are used in distant intercellular communication through RNA signals; they also signal the aforementioned pathogenic materials, mediating AD progression [10,11,12]. The EXO-based cargo delivery system is thought to be mediated by endocytosis, where EXOs release their cargo after entering the targeted cell and may proceed to other physiological and pathological functions [13].

Being spherically shaped nanoparticles ranging from 30 to 150 nm [14] and structurally similar to nanoliposomes, EXOs are being implemented, in nanomedicine, as safe, non-immunogenic drug-carrying vehicles [9,10]. Additionally, the hydrophobic nature and low water solubility of EXOs are the underlying factors enabling them to cross challenging barriers such as the blood–brain barrier (BBB) [15,16]. Moreover, EXOs were tested as successful delivery systems for therapeutic drugs, proteins, and genetic material, including nucleic acids and their derivatives, and thus can interfere with several metabolic paths of AD [9].

EXOs are excreted in the saliva and urine, in either healthy or diseased conditions, making them an accessible and non-invasive diagnostic method, especially in AD and cancers, which require brain or tumor autopsy for diagnosis [10,14]. For instance, urinary exosomal proteins have been implemented as a diagnostic biomarker, especially in urinary tract infections. In 2006, Zhou et al. found higher levels of fetuin-A, a urinary exosomal protein, in patients with acute kidney injury (AKI) [17,18]. Additionally, two years later, Zhou et al. noticed that the activated transcription factor-3 (ATF_3_) was linked to AKI rather than chronic or control cases, implying that both increased exosomal fetuin-A and ATF_3_ can be effective biomarkers in AKI diagnosis [18,19]. Based on this concept, EXOs were implemented as a powerful diagnostic tool for several neurodegenerative disorders. This review does not reiterate previous publications but provides insightful search into the role of the EXOs as pathological, therapeutic, and diagnostic players in AD.

## 2. Exosomes as a Player in AD Pathogenesis

The outlook for EXOs has been completely changed from being simply cell waste to being a natural, multi-tasking carrier that can assimilate and transport large molecules such as lipids, proteins, and nucleic acids. EXOs are increasingly recognized as a vital communication and signaling mechanism in the body, in either healthy or diseased conditions, and play a significant role in transmitting and propagating protein aggregates in disease, especially proteins involved in neurodegenerative diseases [20].

**Figure 1:**
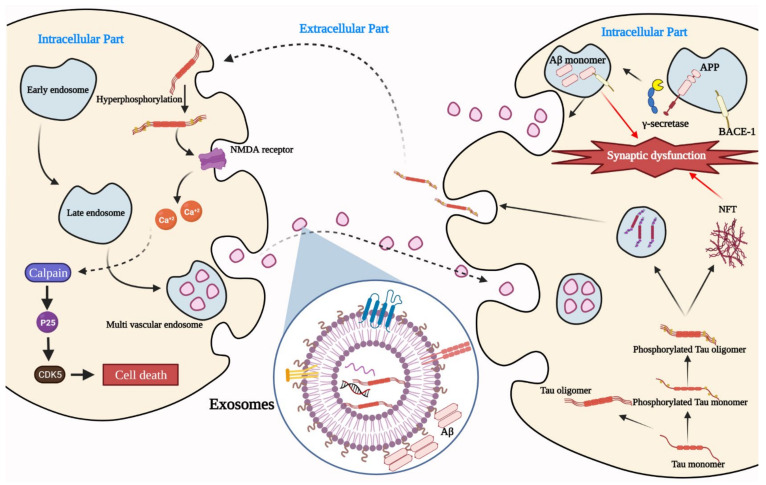
The pathological role of exosomes in AD. NMDA: N-methyl-D-aspartate receptor, CDK5: cyclin-dependent kinase 5, P25: protein 25, Aβ: amyloid-beta, APP: amyloid precursor protein, NFT: neurofibrillary tangles, BACE-1: beta-site amyloid precursor protein cleaving enzyme 1. 636 × 408 mm (118 × 118 DPI).

Figure 1 illustrates how EXOs can carry Aβ oligomers and tau protein, whose accumulation is responsible for causing AD [21,22]. Aβ is the principal component of amyloid plaques, extracellular deposits detected in AD patients’ brains. The plaques consist of a tangle of Aβ oligomers and amyloid fibrils forming regularly arranged aggregates [23]. Recent studies prove that soluble Aβ oligomeric forms can be a potential player in AD progression [24]. Aβ peptides are proteolytic derivatives of APP; this process can take place in a different position in the cell and is regulated by the endosomal pathway, which is likewise crucial for forming EXOs [25].

AD-associated peptides were first discovered in EXOs while tracking APP cleavage events [26]. EXOs have consistently contained APP and C-terminal fragments of APP (CTFs-APP) and numerous proteases involved in APP processing [27]. The active cleavage of APP triggered by β-and γ-secretase within early endosomes yields Aβ to the MVBs. A small fraction of the Aβ peptide was found to be sorted into intraluminal vesicles in MVBs, resulting in the export of Aβ in EXOs [20]. Although EXOs reconstitute an essential route for the clearance of Aβ from the cell, they might pose a risk to the nearby cells by increasing Aβ aggregation potential [28]. Another study by Sinha et al. 2018, provided supportive evidence by showing Aβ oligomer-rich EXOs in Alzheimer’s brains and thus proved that EXOs could act as transfer vesicles among neurons [29].

Tau proteins are synthesized from a single gene called microtubule-associated protein tau (MAPT) and are abundant in the distal region of nerve cells for stabilizing microtubules. Tau phosphoprotein is significantly involved in promoting tubulin assembly into microtubules and stabilizing their structure. The adult human brain typically contains 2–3 moles phosphate/mole of tau protein. Hyperphosphorylation of tau impairs tau’s biological activity, and brain tau is 3-to-4-fold more hyperphosphorylated in AD [30]. Therefore, when tau proteins become defective, microtubule stabilization fails [30,31,32].

Further, when hyperphosphorylated, tau proteins can lead the helical and straight filaments to form NFT; these tangles can accumulate as a toxic cluster in the critical regions for learning and memory [33]. The spreading of these aggregates during the early stage of AD is still poorly understood. Asai et al., 2015 suggested that microglia help to spread the harmful tau proteins through an EXO-based mechanism that releases tau proteins in AD [34,35].

Inflammation arising from microglia- and astrocyte-secreted cytokines, as well as the free radicals, are connected to the progression of AD [36]. Further, EXOs possess a significant role in neuroinflammation because they are a natural nanocarrier of neurotoxic inflammatory molecules and mediate intercellular communication between cells; they also release Aβ, thus accelerating amyloid plaque formation and facilitating the progression of inflammation in brain cells [36,37]. It is believed that Aβ plaques induce downstream effects at the cellular level, such as oxidative stress, microglial activation, local inflammation, and tau protein hyperphosphorylation, leading to cell death and synaptic signaling dysfunction [38,39,40].

## 3. Exosomes and Blood–Brain Barrier

The role of the BBB is to maintain homeostasis and act as a protective shield against harmful threats [41], holding back almost all of the small molecules and 100% of the large ones [42]. The structure of the BBB is a complicated system made up of various cells, including astroglia, perivascular macrophages, basal lamella, pericyte, and endothelial cells, besides the presence of multidrug transporters, efflux pumps, ectozymes, and endozymes [43,44]. For these reasons, lipid-mediated diffusion is the key to transporting only liposoluble molecules with a diameter of less than 400 Da and a few hydrogen bonds across the BBB [45].

Improving CNS exosomal delivery through bio-engineered molecules and surface modification has acquired growing interest [46]. For example, modified surface proteins with overexpressed Lamp2b bound to the neuron-specific rabies virus glycoprotein (RVG) peptide were found to enhance CNS penetration and targeting capabilities [47].

Due to the nanometer-sized diameter and permeability, EXOs can facilitate the penetration of the BBB and therefore engage in various signaling events, providing insight into the development and management of brain disorders [48]. Current data reveal the possibility that EXOs pass the BBB through different mechanisms other than diffusion, mainly through the different transcytosis, efflux, and carrier-mediated transport mechanisms [47]. Yang et al., 2015 proposed the potential role of the exosomal membrane tetraspanins in passing to the brain [49]. They studied EXOs derived from four brain cells whose surfaces carry CD9, CD81, and CD63 tetraspanins. Notably, tetraspanin proteins CD9 and CD81 showed comparable levels in the four types; however, significantly elevated levels of CD63 were found in the brain endothelial cell-derived EXOs, resulting in enhanced CNS penetration compared to the other three exosomal types. Tetraspanins organize the multimolecular membrane complexes, cell adhesion, fusion, signaling, and metastatic processes [50,51], and they were also reported to serve as a facilitative component in infection processes, promoting viral penetration and exit [52].

## 4. Exosomes as a Drug Carrier

Polymeric nanoparticles, along with liposomes, have been traditionally implemented in drug delivery systems for various health conditions; however, liposomes are still incapable of alleviating their immunogenicity, short circulating time, stability issues, and toxicity. The same concerns exist for polymeric nanoparticles; although they may have better stability levels than liposomes, their biocompatibility, in addition to long-term safety profiles, remains a concern. On the other hand, EXOs possess valuable characteristics, such as a long half-life, targeting capabilities, biocompatibility, and almost non-immunogenicity, making them the preferred choice compared to liposomes or polymeric nanoparticles [10]. The concept of engaging EXOs in drug delivery has emerged from their role as an intercellular communication mediator since they enable cellular transfer for foreign substances and cargo, such as proteins, mRNAs, miRNAs, and lipids [53]. EXOs may have a multitude of functions in AD treatment.

Multipluripotent mesenchymal stem cells (MP-MSCs) are connective tissue-derived adult stem cells, including adipose tissue and bone marrow. EXOs derived from these cells (MSC-EXOS) have emerged as promising vehicles for pharmaceuticals and play a neuroprotective role in traumatic brain injury and neurodegenerative illnesses by supporting functional recovery while inhibiting apoptosis and neuroinflammation [36,54].

Chen et al., 2021 illustrated the curative mechanism of MSC-EXOs and proposed a cell-free MSC-EXO-based therapy method for treating AD. Results showed that the MSC-EXO therapeutic approach reduced Aβ expression, cleared brain glucose metabolism, and improved cognitive function and neuronal plasticity [55].

### 4.1. Isolation of Exosomes

EXOs and EXO-like vesicles have been isolated from animal and plant sources using a variety of methods. Table 1 presents the advantages and disadvantages of different exosome separation methods, including the following:

#### 4.1.1. Ultracentrifugation

Ultracentrifugation (UC) is the gold-standard process for isolating and purifying EXOs and EXO-like particles because of its non-complexity and low cost; although the procedure improves the purity, it decreases the yield of separated EXOs [56,57]. To address this defect, efforts have been made to scale up the EXO isolation approach, conserving greater purity and yield. Hence, ultrafiltration was introduced to separate biomolecules based on their sizes, providing a faster solution to UC that increases the EXO yield. Nevertheless, this method contains an additional step, leading to contamination and rising production costs [56,57,58].

#### 4.1.2. Size-Exclusion Liquid Chromatography

Size-exclusion liquid chromatography (SEC) helps to isolate EXOs based on size differences; it is suitable with purification from serum or plasma, does not require pelleting EXOs at high speeds, and produces a highly purified product [59]. However, SEC must be used in conjunction with other methods to avoid possible interference between sample and lipoproteins and the possibility of protein aggregation [60].

#### 4.1.3. Immuno-Isolation

The immuno-isolation technique uses antibody-coated magnetic beads that target proteins on the EXO surface, thus ensuring high-purity isolation. Nevertheless, the lack of knowledge on exosomal structures, surface components, and large-scale issues are still challenging drawbacks for this method [56,61].

#### 4.1.4. Microfluidic Isolation

Microfluidic devices, a new tool composed of microchannels and microchambers, approved for many applications, are used in the biological sciences and engineering to isolate, detect, and analyze micro-and nano-particles.

Microfluidics emerged as an advanced technique for isolating EXOs, offering several advantages, such as providing high-purity separation and being economical and less time-consuming. These advantages rely on the device design having a large surface-to-volume ratio, reducing the needed volume for valuable samples, and accelerating the reaction speed by simultaneously carrying out numerous stages [57,60].

#### 4.1.5. Paper-Based Immunoaffinity

Paper surface modification can be achieved utilizing a chemical conjugation technique to create a paper-based immunoaffinity system. The capture molecule is chosen as an antibody with a high affinity for a specific EV; therefore, this approach is quick and straightforward and uses a minimal sample amount [60,62].

**Table 1:** Advantages and disadvantages of different isolation methods for exosomes.

Isolation Method	Advantages	Disadvantages	Ref
UC	Straightforward Low-cost	Low yield Time-consuming Prone to contamination	[56,57,58,60]
SEC	Nondestructive, reproducible Size-related separation of EXOs Suitable for various biological samples High-purity yield	Protein aggregation Interference between sample and lipoproteins	[59,60]
Microfluidic Isolation	High-purity isolation Economical Deals with minute sample volumes Time-efficient	Highly complex May result in irreversible destruction of isolated exosomes	[57,60]
Paper-Based Immunoaffinity	Ultra-pure yield Requires minimal sample amount Simple and fast	The specificity of the used antibodies governs the selection process Low yield	[60,62]
Immuno-Isolation	High-purity isolation	Knowledge of the exosomal surface components is a challenge in this method Scale-up	[56,61]

### 4.2. Drug Loading to Exosomes

**Figure 2:**
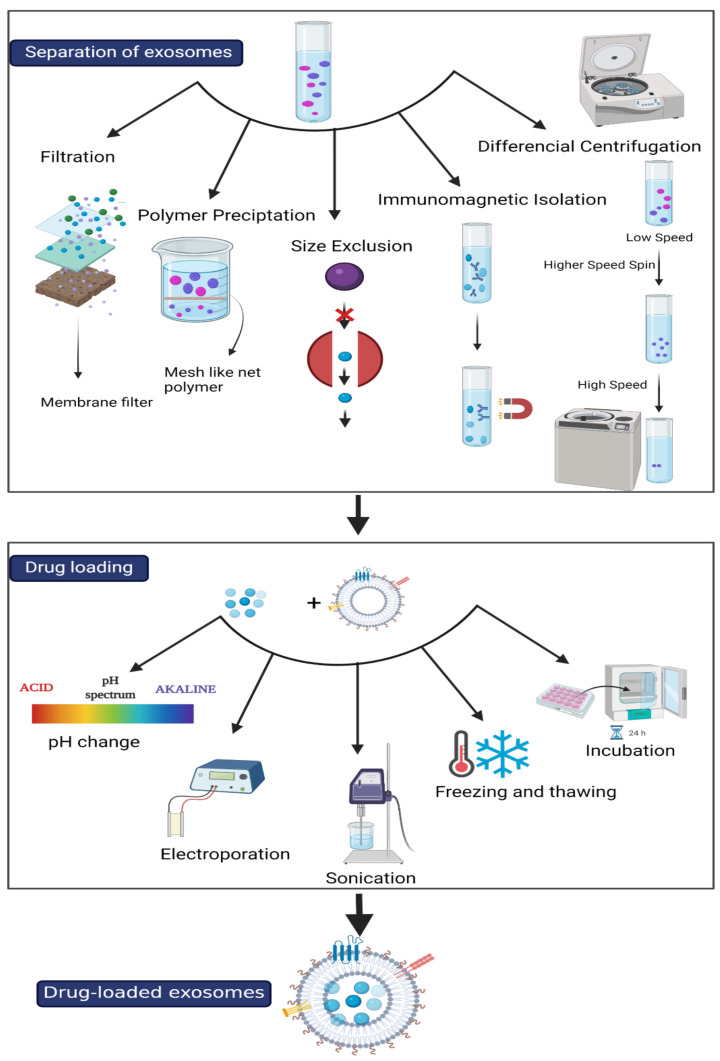
The separation and drug loading techniques for exosomes. 475 × 872 mm (118 × 118 DPI).

Prior to cargo loading, EXOs are recommended to undergo a miniature cargo off-loading process, which helps to free up sufficient space ahead of the actual cargo loading process. The off-loading process should operate under controlled measures to ensure intact structural integrity, which provides efficient encapsulation as an optimal drug delivery system [56]. Exosomal cargo loading is conceivable through two main strategies: passive and active, as indicated in Figure 2. Applying these strategies will affect both the loading efficiency and overall system efficacy [63].

#### 4.2.1. Passive Loading

Passive cargo loading includes the incubation of drugs with EXOs or with donor cells. It is mediated through the simple diffusion of the drug into EXOs along its concentration gradient. However, this procedure can represent a significant setback for the loading efficiency as it requires loading hydrophobic drug molecules only to diffuse through EXOs and the host bilipid membrane [56,63]. Moreover, the incubated drug with the host cells is not a reliable approach for their low yield, despite its observed benefit in releasing EXOs carrying the active pharmaceuticals (APs), as demonstrated with Paclitaxel in mesenchymal stromal cells [56,64]. 

#### 4.2.2. Active Loading

On the other hand, active loading involves subjecting EXOs to shear stress that temporarily affects the membrane permeability, resulting in APs’ diffusion into the vesicle [56]. These methods take advantage of the shear stress mediated by the action of different external factors, such as electrical pulses in electroporation, probes in sonication, and temperature in both extrusion and freeze–thaw operating cycles. While there is a reported variation in loading efficiency for different drug types, the highest loading efficiency is usually observed in sonication, extrusion, and permeabilization with saponin detergent methods [14]. Moreover, Fuhrmann et al., 2014 reported saponin-assisted loading as 11-fold more efficient for hydrophilic porphyrins than passive loading [65]. Given that each method operates differently, there should be a consideration of the nature of the intended loaded matter—a drug molecule, siRNA or miRNA, protein, or an enzyme—when choosing the ideal loading method. However, it should be noted that electroporation is commonly used for loading siRNA or miRNA segments, while sonication is preferentially applied for loading drugs or proteins [14]. The major drawback of the active loading technique is that it affects the targeting abilities of EXOs by damaging their structural integrity and losing their surface proteins [56]. Akuma et al., 2019 suggested that combining both loading approaches can modulate their drawbacks [56].

### 4.3. Role of Exosomes in AD Treatment

**Figure 3:**
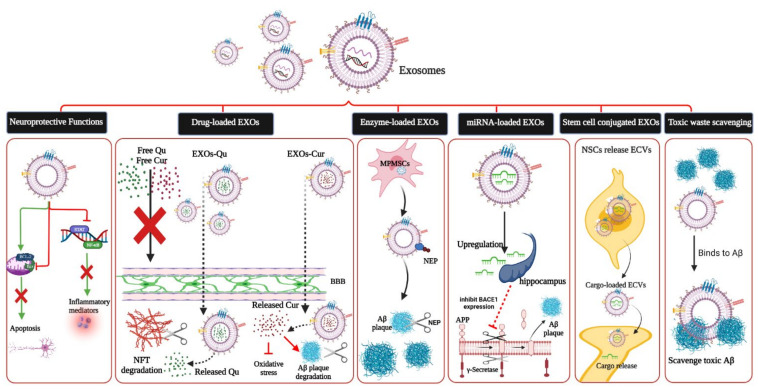
Therapeutic application of exosomes in AD. BCL2: B-cell lymphoma 2, BaX: BcL-2-associated X protein, STAT: signal transduction and activator of transcription protein, NF-κB: nuclear factor kappa-light-chain enhancer of activated B-cells, QU: quercetin, Cur: curcumin, BBB: blood–brain barrier, NFT: neurofibrillary tangles, MP-MSCs: multipotent mesenchymal stem cells, NEP: neprilysin, BACE-1: beta-site amyloid precursor protein cleaving enzyme 1, APP: amyloid precursor protein, Aβ: amyloid-beta, NSCs: neural stem cells, ECVs: extracellular vesicles. 1081 × 540 mm (118 × 118 DPI).

Due to their RNA transport capacity, stability in body fluids, and capability of crossing the BBB, EXOs can be used as carriers to deliver nucleic acid fragments, such as miRNA and siRNA, for AD treatment [47]. Moreover, neuron-derived EXOs can provoke conformational modifications to extracellular Aβ, turning them into non-toxic fibrils, promoting microglia uptake [28]. EXOs can be applied in treating AD in different ways, as follows (Figure 3).

#### 4.3.1. As a Drug Delivery Vehicle

The use of EXOs to deliver APs has a variety of advantages. EXOs generated from a particular donor patient’s cells are less immunogenic and thus have more biocompatibility and less toxicity than manufactured medication carriers. Furthermore, EXOs can penetrate tissues, disperse into the blood, and possess a unique capacity to pass the BBB, making them perfect carriers for drug and genetic components to treat neurological disorders [66,67].

Quercetin (Que), a naturally occurring cognitive enhancer known for its neuroprotective and anti-inflammatory actions [68], has been reported to suppress tau-mediated AD pathophysiology and, to a significant extent, Aβ plaques by inhibiting amyloid production [69]. However, further clinical applications of Que were stopped because of their poor solubility, reduced systematic concentration, and inability to penetrate the BBB [70,71].

Qi et al., 2020, developed EXOs loaded with Que (EXO-Que), which was applied to okadaic acid-induced AD in rats to improve Que bioavailability and brain targeting and potently enhance cognitive function. Furthermore, when compared to free Que, EXO-Que decreased insoluble NFT formation and constrained the cyclin-dependent kinase 5-mediated phosphorylation of tau, ensuring its therapeutic potential for AD treatment [70].

To enhance the delivery of therapeutic bioactive chemicals, EXO-like liposomes (EXO-liposomes), newly modified liposomes, were developed to mimic the natural EXOs; they overcome the limitations of EXOs, such as heterogeneity and low productivity [72]. To test the efficiency of the new model, Curcumin (Cur), which possesses anti-inflammatory action and the potential capacity to halt oxidative stress and Aβ deposits [73], was chosen to be loaded in this EXO-liposome model. The new model showed promising results as the encapsulation proficiency of Cur was up to approximately 94%, Cur-loaded liposomes showed no cytotoxic activity (up to 20 μM Cur and 200 μM of exo-liposomes), and the overall significance was to mitigate the induced oxidative stress in neuronal cells, indicating their neuroprotective effect [72].

#### 4.3.2. Enzyme Delivery

The stem cell secretome consists of various biologically active substances. EXOs are among the stem cell secretome, where exciting findings showed that EXOs derived from dental pulp stem cells (DPSCs) [74], bone marrow stromal cells (BMSCs), and adipose-derived stem cells (ADSCs) contain neprilysin and insulin-degrading enzyme [75,76], whose functions are to degrade Aβ [77]. The amount of neprilysin in EXOs secreted by DPSCs was relatively higher than EXOs from BMSCs and ADSCs. Moreover, these EXOs were capable of Aβ_1–42_ degradation and attenuated their neurotoxic effects in SH-SY5Y neuroblastoma cells in vitro [75].

#### 4.3.3. miRNA Delivery

Non-coding regions on RNA strands (introns) such as miRNA [78] are regulatory codons involved in protein expression. Furthermore, any alteration in their expression can propagate disease conditions due to dysregulated, non-functioning codons, as in the case of many neurodegenerative disorders [79]; thus, they can be incorporated into AD gene silencing treatment regimens [80].

Alvarez-Erviti et al., 2011 were the first to test the potential of gene therapy in AD using animal models (mice). Purified RVG-targeted EXOs were loaded with exogenous siRNA using electroporation. Under such circumstances, a significant downregulation in protein expression and reduction in Aβ plaques in mice with AD were achieved, validating EXO-based gene therapy in treating AD. In another study, the neuronal–glial delivery of miR-124a through EXOs resulted in the upregulation of the excitatory amino acid transporter 2 (EAAT2, rodent analogue GLT1) on the astrocytes’ surface, enhancing glutamate uptake, which is critical for preserving intact cognitive function in this case [81,82].

#### 4.3.4. Toxic Waste Scavenging

EXOs can operate as effective Aβ scavengers by binding to Aβ through enriched glycans on glycosphingolipids on the membrane surface, suggesting a role for EXOs in Aβ clearance in the central nervous system. Improving Aβ clearance by EXO administration offers a new medicinal pipeline for AD [83]. EXOs from the neuronal genetically modified neuroblastoma cell line (N2a cells) can neutralize Aβ-induced disruptions in synaptic plasticity and prevent Aβ-induced neuronal apoptosis [84,85].

Interestingly, Yuyama et al., 2014, declared the role of EXOs as Aβ scavengers, mediated through Aβ-glycan and EXO-glycosphingolipid attachment, leading to a significant reduction in Aβ count and therefore the mitigation of AD pathophysiology [83].

#### 4.3.5. Neuroprotective Effects

Glutamate excitotoxicity has been linked to several neurological illnesses and implicated as a pathogenic pathway causing neuronal cell death. Currently, stem cell transplantation is becoming a promising therapeutic option for neurological disorders. Administration of EXOs generated from MSCs could improve various neurologic conditions by restoring the biomolecules necessary for non-functional cells. Human adipose-derived MSCs secreted EXOs that exerted direct neuroprotective effects by preventing neuronal apoptosis, thus supporting the regeneration and repair of the CNS and hence restoring bioenergy after exhaustion due to glutamate excitotoxicity [86].

NF-κB is found almost in all cell types and is involved in cellular signaling and stimuli such as cytokines, stress, free radicals, oxidized-LDL, and microbial antigens [87]. Alterations of NF-κB have been linked to tumorigenesis, inflammatory and autoimmune diseases, septic shock, and viral infection. NF-κB has been associated with the formation of synaptic plasticity and memory as well [88]. Treatment with MSC-derived EXOs was shown to control glial cells and diminish the levels of inflammatory mediators regulating the NF-kB pathways [89].

Previous investigations revealed that EXOs could have the ability to reduce brain Aβ-protein by being ingested by microglia. They can transmit neuroprotective chemicals among cells as well [90,91,92].

## 5. Diagnostic Role of Exosomes

The great diagnostic potential of EXOs lies in the fact that these nanosized vesicles have a specific profile of biomolecule content from the cell of origin, similar to proteins, nucleic acids, and lipids; they act as a “fingerprint” or a “signature” of parent cells and can reflect the pathological conditions when changes occur in their content [79,93]. Moreover, these EXOs preserve high stability, protecting their content; hence, patient samples can be stored for further biomarker analysis. EXOs are easily accessible in almost all body fluids, including the blood, urine, cerebrospinal fluid (CSF), saliva, synovial fluid, breast milk, semen, amniotic fluid, ascites, and lymph [94,95,96].

EXOs include several tumor-related proteins in lung cancer, such as epidermal growth factor receptors (EGFR), KRAS, and extracellular metalloproteinase inducer. The possible exosomal markers in non-small-cell lung cancer (NSCLC) were proposed for CD91, CD317, and EGFR. Exosomal EGFR is one of the NSCLC membrane-bound proteins. In particular, 80% of the EXOs isolated from non-small-cell lung cancer tissues were EGFR-positive, as Huang et al., 2014 reported [97].

The exosomal protein difference was recently evaluated utilizing a triple SILAC quantitative proteomic approach expressed in normal bronchial epithelial cells and NSCLC [98]. They discovered that NSCLC-EXOs are abundant in cell signal proteins and extracellular matrix remodeling for cell adherence. Seven-hundred-twenty-one exosomal proteins generated from three cell lines were identified and quantified. Among the enriched proteins involved with signal transduction, EXOs, EGFR, SRC, and downstream effectors such as GRB2 and RALA levels were increased. In addition, MET receptor, RAC1, and KRAS proteins were elevated as well.

Sandfeld-Paulsen examined 49 exosomal membrane-bound proteins in a cohort study of 276 NSCLC patients, and this demonstrated that nine proteins have potential as prognostic markers in NSCLC. In particular, the study showed that rising NYESO-1, EGFR, and PLAP concentration levels are predictive indicators of poor prognosis [99]. A proteomic study of EXOs extracted from human pleural malignancy has shown that the vesicles included previously identified components of other EXOs, such as MHC proteins Class I and II, thermal and cytoskeletal proteins, and signal transduction proteins.

Moreover, the recent finding of circular RNAs (circRNAs) in serum EXOs indicates a new and possibly helpful technique to diagnose non-invasive malignancy. RNA-sequencing tests were conducted among three pairs of patients with NSCLC and controls [100].

For 218 of the 467 mature microRNAs examined, microRNA from ovarian tumor cells and EXOs from similar individuals were positive [101]. The amounts of eight particular microRNAs between cellular and exosomal microRNAs were comparable (exhibiting correlations from 0.71 to 0.90). While both individuals with benign and ovarian cancer may have possessed EpCAM-positive EXOs, exosomal microRNA in ovarian cancer patients showed comparable patterns that were substantially different from profiles found in benign disease. The authors were unable to identify exosomal microRNA in normal controls. These findings indicate that the profile of circulating tumor EXOs by microRNA and different biomarkers may be utilized for biopsy profiling as replacement diagnostic indicators to expand their usefulness in asymptomatic population screening, as presented in (Table 2) [101].

### 5.1. Role of Exosomes in AD Diagnosis

The changes in the size and concentration of EXO-derived biomarkers demonstrate their potentially high diagnostic value for AD or mild cognitive impairment (MCI) [110,111]. Wang et al., 2018 found that the tau aggregate N2a cell model preferentially discharged aggregates through EXOs since they contain more tau than the N2a cell [112]. They discovered that tau aggregates that include EXOs might encourage the aggregation of tau in cultured cells. This would explain why tau’s illness spreads hierarchically and is not closely dependent. If the tau aggregates could be released along the axon shaft, other processes would be anticipated to prevent the tau aggregates’ absorption by neighboring neurons.

#### 5.1.1. Blood Samples

Fiandaca et al., 2015 [113] measured the levels of pathogenic proteins in the blood of patients with AD, including total tau, P-T_181_-tau, P–S_396_-tau, and Aβ_1-42_ in neuron-derived EXOs (NDEs). The researchers observed significantly elevated pathogenic blood proteins in preclinical individuals up to 10 years before being diagnosed with AD. Moreover, levels of EXO-derived Aβ_1–42_ increased from non-symptomatic to AD; thus, they can be a potential disease progression biomarker.

#### 5.1.2. Plasma Samples

More recently, in 2020, Gu et al. [114] measured the levels of Aβ_42_, P-T_181_-tau, P-S_396_-tau, and other inflammatory biomarkers, including interleukin 6 (IL-6) and matrix metalloproteinase-9 (MMP-9), in the plasma NDEs of AD and control patients. There was an overall increase in the expression of Aβ_42_, P-T_181_-tau, and metalloproteinase-9 (MMP-9) in AD patients compared to controls, whereas no difference was detected in the P-S_396_-tau and IL-6 levels in plasma NDEs.

Another study conducted in 2016 by Abner et al. [115] investigated the plasma NDE levels of P-T_181_-tau, P-S_396_-tau, Aβ_1-42_, repressor element 1-silencing transcription factor (REST), and neurogranin (Ng) in both AD patients and cognitively intact subjects (CIS) at 3- to 11-year intervals. Study findings showed that the expression levels of plasma P-T_181_-tau, Aβ_1-42_, and REST increased over time with age, while Ng decreased and there was a slight change in the P-S_396_-tau level over the same interval. At the same time, Winston et al. [116] applied the previously mentioned plasma NDE biomarkers to predict the switch from MCI to AD and compared these levels with controls. Combining the use of elevated biomarkers such as Aβ_1–42_ levels in NDEs and lower olfactory function scores measured using sniffing sticks (SS-16) in patients with MCI showed their predictive potential in AD development [117]. Furthermore, AD biomarkers in NDEs, including Aβ and P-tau, were significantly increased in Down syndrome individuals [118,119].

On the other hand, synaptic proteins were harnessed as a biomarker for AD since synapse dysfunction occurs early and has been highly correlated in AD [120,121]. Jia et al. [122] measured the levels of growth-associated protein 43 (GAP43), Ng, synaptosome associated protein 25 (SNAP25), and synaptotagmin-1 (Syt-1) in both blood and CSF NDEs, which were highly inversely correlated. The study revealed that the combination of exosomal GAP43, Ng, SNAP25, and Syt-1 could be an early biomarker candidate for AD in 5 to 7 years before the onset of cognitive impairment. Additionally, synaptophysin, synaptopodin, Syt-1, GAP43, and Ng showed decreased levels of plasma NDEs in patients with AD and frontotemporal dementia, which were lower than controls.

Moreover, Goetzl et al. extracted neural cell CSPG4 EXOs (CSPG4Es) from human plasma, using successive anti-CSPG4 and an anti-platelet growth factor mAbs for immunoadsorption [123]. The hepatocyte growth factor, FGF-2, -13, and IGF-1 were part of the CSPG4E extracts and enhanced neuronal survival and function. In the CSPG4Es of the 24 healthy controls, HGF, FGF-13, and IGF-1 were up to seven-times higher than in neural generated EXOs and up to eight-times higher in astrocyte-originated EXOs. All growth factors showed reduced mean CSPG4E in mild Alzheimer’s (*n* = 24 patients) compared with cognitively normal age- and sex-matched controls (*n* = 24). The mean CSPG4E levels of all growth factors were also considerably decreased for 15 people with moderate AD (AD2) dementia compared to their preclinical stage 3 to 8 years earlier (AD1).

#### 5.1.3. Serum Samples

In addition to proteins, several studies demonstrated the potential application of exosomal miRNAs as biomarkers in AD, as presented in Table 3**.** In 2018, Yang et al. [124] measured the serum expression levels of miR-135a, -193b, and -384 with a real-time quantitative reverse transcriptase-PCR (qRT-PCR) method; the findings presented an increased expression level of both miR-135a and miR-384 from MCI and AD groups compared to the control group. On the other hand, the exosomal miR-193b level in the serum of MCI and AD patients was downshifted. Furthermore, the three exosomal miRs applied in the Yang study could discriminate between different dementia types, including AD, Parkinson’s disease with dementia, and vascular dementia.

Dong et al., 2015, identified the serum EXO miRNAs, such as miR-31, miR-93, miR-143, and miR-146a, using Solexa sequencing and qRT-PCR for AD diagnosis [125]. These four miRNAs were markedly decreased in the AD patients compared with the control subjects.

#### 5.1.4. Cerebrospinal Fluid Samples

EXOs in the CSF have recently been identified as prospective biomarkers for central nervous system (CNS) disease [126]. In addition to blood EXOs, a strong correlation was found between CSF exosomal biomarkers and AD diagnosis since Aβ_42_, T-tau, and P-T_181_-tau differed significantly in AD patients and those with MCI and controls [127]. It is widely known that CSF Aβ_42_, total tau, and p-tau181 are AD markers [128,129]. Using CSF biomarkers may distinguish between Alzheimer’s and other dementias in cognitively challenged individuals [130]. It also predicts future cognitive deterioration in cognitively normal individuals with a 5–10 year follow-up [131,132]. While absolute CSF Aβ_40_ and Aβ_38_ levels have limited diagnostic value in AD, the Aβ_42_/Aβ_40_ or Aβ_42_/Aβ_38_ ratios have several advantages [133]. In clinical trials of amyloid-based treatments, the CSF Aβ_42_/Aβ_40_ and Aβ_42_/Aβ_38_ ratios may be a better predictor of target engagement than CSF Aβ_42_ alone [134,135].

#### 5.1.5. Saliva Samples

Rani et al. [136] recently used a novel technique based on nanoparticle tracking analysis as a non-invasive and low-cost method to determine the salivary EXO concentration. This study suggested that salivary exosomal levels can be significantly increased in patients with cognitive impairment and AD.

#### 5.1.6. Urine Samples

EXOs contain almost all the biomolecules that come from the parental cells. EXOs can be found in physiological fluids, including urine, which can be collected easily and non-invasively. Therefore, urine EXOs are excellent and the most common candidates for liquid biopsy [137]. Sun et al. [138] measured the levels of Aβ_1–42_ and P-S_396_-tau in urinary EXOs in a pilot study. This study suggested that these pathological proteins’ levels in urinary EXOs were significantly elevated in AD patients compared to control subjects.

**Table 3:** Examples of exosomal miRNA biomarkers in AD.

Biofluid	Increasing	Decreasing	Ref
Serum		miR-193b	[139]
	miR-193b *		[140]
	miR-135a and miR-384		[124,141]
		miR-193b	[124]
	miR-128		[142]
Plasma		miR-193b	[139]
	miR-92a-3p, miR-181c-5p and miR-210-3p		[143]
CSF		miR-193b	[139]
	miR-135a		[141]
	miR-193b *		[140]

* This miRNA was found in ABCA1-labeled exosomes.

## 6. Future Outlook

EXOs have attracted significant attention during the past decade because of their substantial role in communication between cells and their ability to reflect the real-time state of the original cells. Therefore, EXOs have acquired increasing importance in clinical practice as a diagnostic and therapeutic tool. Due to EXOs’ heterogeneity in source, size, and content, there are still some challenges in the isolation of EXOs. To overcome these problems, recently, microfluidics technologies were developed to enable the handling or manipulation of small volumes of liquids in microchannels, resulting in high efficiency, automation, and reducing the risk of cross-contamination; thus, the use of microfluidic devices represents a promising technique for EXO separation from biofluids and as a diagnostic biomarker in liquid biopsy [144]. Moreover, the presence of EXOs in biofluid samples (blood, urine, saliva, cerebrospinal fluid) can be employed in diagnostic innovation as potential candidates for liquid biopsy.

A liquid biopsy is a medical tool that can be used in conjunction with diagnostic protocols, molecular monitoring, or disease staging for various diagnostic purposes. Compared to liquid biopsy, traditional solid biopsy has significant limits due to the exponential evolution. Therefore, a liquid biopsy must be introduced into clinical practice to eliminate invasive procedures and encourage more precise medical intervention [145].

One of the essential techniques realized by microfluidic technologies and the use of high-level microfabrication techniques traditionally developed for mass production in micro- and nanoelectronics is Lab-on-a-Chip (LOC) technology. The LOC technique offers customizable opportunities for EXO isolation based on many physicochemical criteria, such as immunochemistry, sieving, acoustic wave, and field-flow fractionation [146]. Although none of the microfluidic approaches for neurodegeneration reviewed so far have used EXOs, they have involved the most commonly used biomarkers for diagnosing neurodegenerative disorders. Despite the promise of microfluidic platforms for protein detection, they still cannot detect low protein concentrations in the blood because of the rapid metabolism of soluble proteins in circulation [147].

A microfluidic device made of polydimethylsiloxane (PDMS) and functionalized with antibodies against CD63, a common EXO antigen, called “ExoChip” was used by Kanwar et al., 2014, by which EXOs were stained with a fluorescent carbocyanine dye (DiO) that marked them explicitly, and EXOs were quantified using a conventional plate reader. The immuno-affinity technique was used to isolate EXOs in ExoChip [148]. Microfluidic systems can produce EXO mimics; however, these mimetics have a structure similar to cell-derived EXOs. Bottom-up EXO mimetics always include chemical components that are likely to cause biocompatibility difficulties. Therefore, they are simply not suitable to be employed in therapeutic or clinical applications. More research has to be conducted to address these concerns in order to comprehend the cellular absorption process, targeting efficiency, stability, in vivo dynamics, and potential side effects of EXO mimetics. Organ-on-a-Chip devices that can mimic in vivo physiological microenvironments are predicted to be a valuable solution for evaluating EXO mimetics’ cytotoxicity to speed up such research and the development of EXO mimetics [149].

One of the latest technologies that has appeared recently is the Fab-TACS EXO Isolation Kit. This isolation technology allows the rapid isolation of pure and unlabeled EXOs, free of antibodies and magnetic beads. Development is still ongoing to reach more accurate isolation and more specific cell and tissue targeting due to the differences in the types of EXOs and the cargo that they carry in each organ [150].

## 7. Conclusions

The applications of extracellular vesicles are diverse and numerous, but they are still in the infancy stage of development due to the limitations related to their manipulation. Reaching an advanced stage and developing techniques that deal with the EXOs in an ideal way for accurate separation, identification, and diagnosis, in various clinical applications, require extensive research and more experiments. Recent work on exosomes identified various aspects pertaining to their roles in the pathogenesis, diagnosis, and treatment of AD. Their prominent role in transporting proteins, RNA, and miRNA in distant intercellular communication paved the way to their implementation as a vibrant drug delivery system in AD treatment. They carry various cargo, ranging from drugs such as Quercetin and Curcumin to even enzymes such as neprilysin and insulin-degrading enzyme. Paying more attention to the microfluidic techniques and EXOs will lead to important advances in the field of clinical diagnostics and therapeutics. When technically and medicinally optimized, these techniques will overcome several obstacles and achieve unlimited goals: long circulation, high biocompatibility, high penetration through difficult barriers, protection of cargo from degradation, compartmentalization, and cell-/tissue-specific targeting.

## Figures and Tables

**Table 2:** Examples of exosomal biomarkers with diagnostic potential in cancer.

Diseases	Exosomal Biomarkers	Ref
Prostate cancer	Survivin, PCA-3, TMPRSS2:ERG, PSA, miR-141, and miR-375	[102,103,104,105]
Esophageal squamous cell cancer (ESCC)	miR-21 and miR-1246	[106,107]
Breast cancer	miR-21	[108]
Colorectal cancer	mRNAs	[109]
